# Does prevention-focused integration lead to the triple aim? An evaluation of two new care models in England

**DOI:** 10.1177/1355819620963500

**Published:** 2020-10-27

**Authors:** Jonathan Stokes, Vishalie Shah, Leontine Goldzahl, Søren Rud Kristensen, Matt Sutton

**Affiliations:** 1 Research Fellow, Health Organisation, Policy, and Economics, Centre for Primary Care and Health Services Research, University of Manchester, UK; 2 Research Associate, Health Organisation, Policy, and Economics, Centre for Primary Care and Health Services Research, University of Manchester, UK; 3 Associate Professor, EDHEC Business School, France; 4 Senior Lecturer, Faculty of Medicine, Institute of Global Health Innovation, Imperial College London, UK; 5 Associate Professor, Danish Centre for Health Economics Research, University of Southern Denmark, Denmark; 6 Professor, Health Organisation, Policy, and Economics, Centre for Primary Care and Health Services Research, University of Manchester, UK

**Keywords:** integrated care, multimorbidity, new care models

## Abstract

**Objectives:**

To examine the effectiveness of two integrated care models (‘vanguards’) in Salford and South Somerset in England, United Kingdom, in relation to patient experience, health outcomes and costs of care (the ‘triple aim’).

**Methods:**

We used difference-in-differences analysis combined with propensity score weighting to compare the two care model sites with control (‘usual care’) areas in the rest of England. We estimated combined and separate annual effects in the three years following introduction of the new care model, using the national General Practice Patient Survey (GPPS) to measure patient experience (inter-organisational support with chronic condition management) and generic health status (EQ-5D); and hospital episode statistics (HES) data to measure total costs of secondary care. As secondary outcomes we measured proxies for improved prevention: cost per user of secondary care (severity); avoidable emergency admissions; and primary care utilisation.

**Results:**

Both intervention sites showed an increase in total costs of secondary care (approximately £74 per registered patient per year in Salford, £45 in South Somerset) and cost per user of secondary care (£130–138 per person per year). There were no statistically significant effects on health status or patient experience of care. There was a more apparent short-term negative effect on measured outcomes in South Somerset, in terms of increased costs and avoidable emergency admissions, but these reduced over time.

**Conclusion:**

New care models such as those implemented within the Vanguard programme in England might lead to unintended secondary care cost increases in the short to medium term. Cost increases appeared to be driven by average patient severity increases in hospital. Prevention-focused population health management models of integrated care, like previous more targeted models, do not immediately improve the health system’s triple aim.

## Background

Health systems globally are attempting to integrate care in response to demographic changes and economic challenges,^1^ with an increasing emphasis on what has been referred to as the ‘triple aim’,^2^ that is the simultaneous improvement of patient experience and health status while reducing the cost of health care. Much of the evidence for integrated care to date has tended to focus on a small number of interventions aimed at a targeted small group of individuals, typically those with a single condition or at high-risk of hospitalisation.^3^,^4^ Results have rarely met expectations.^5^

More recently there has been greater focus on what has been described as population health management, with integrated care models seeking to take a (geographically defined) whole-systems approach and to improve outcomes for the local population.^6^ This approach targets ‘place’ rather than a specific patient group and tends to place greater emphasis on disease prevention (in the relatively healthy general population) rather than high-risk patient management.

This study examined two novel models of integrated care that were implemented in England and that sought to take a more population health management approach. Specifically, we evaluated their effectiveness in terms of patient experience, health outcomes and costs of care (‘triple aim’), with a particular focus on the prevention-centred aspects of the care model.

### Study setting

The study was part of a pan-European project that sought to compare new models for safe and efficient prevention-oriented health and care systems.^7^ For England, we selected two new care models in Salford and South Somerset, following a set of selection criteria developed as part of the wider project: scientific criteria, primarily focused on the care process itself, requiring that the programmes addressed multimorbidity and met our operational definition of integrated care;^8^ and pragmatic evaluability criteria, availability of data, an ongoing status of the programme during the study period, the transferability to other care settings, and willingness to collaborate with the European project.^9^

The two study sites were part of the wider New Models of Care (Vanguard) programme that was launched in England in 2014 as a means to overcome the traditional boundaries between primary and secondary care and community services to support improvement and integration of services.^10^ Participating sites received government funding and support to pilot population health management approaches from 2015 (until March 2018). Salford and South Somerset were among the nine sites that implemented ‘integrated primary and acute care systems’ (PACS), both seeking to achieve the triple aim.^11^ Table 1 summarises the specific changes that were implemented by each site across their local system.

**Table 1.** Summary of local system changes implemented in Salford and South Somerset models of care.

Domain	Salford: Located in the North West of England, covering a population of about 250,000 people registered in 45 GP practices	South Somerset: Located in the South West of England, covering a population of 115,000 people registered in 19 GP practices
Service delivery	• Integrated contact centre for patient navigation: central ‘single-point-of-contact’ phone hub for patients to contact, including a health coaching component for selected individuals• Multidisciplinary group case management^a^: individualised identification and management of high-risk patients (case management)• Supporting ‘community assets’: investment in local neighbourhood groups and activities, such as sports groups, to encourage community engagement in healthy social behaviours	• Complex care hub^a^: individualised identification and management of high-risk patients (case management) • Enhanced primary care (19 GP practices): non-medical health coaching for self-management of chronic conditions based in GP practices
Leadership & governance	• Salford Together is a partnership between Salford City Council, NHS Salford Clinical Commissioning Group, Salford Royal NHS Foundation Trust (leading role), Salford Primary Care Together and Greater Manchester Mental Health NHS Foundation Trust. • Originally partnership functioned through an alliance contract, but the creation of an Integrated Care Organisation (ICO) in 2016 saw community, social care and mental health services merge to create a single organisational unit (based at the hospital)	• Leadership by Symphony Programme Board, co-located in Yeovil hospital. The Symphony Programme is a collaboration between Yeovil District Hospital NHS Foundation Trust, south Somerset Healthcare GP Federation, Somerset CCG, Somerset Partnership, the voluntary sector in South Somerset (SSVCA) and Somerset County Council. • Formation of accountable care provider organisation, Symphony Healthcare Services Ltd, from acquisition of a small number of initially four GP practices
Workforce	• New ways of working, incorporating multiple specialties (health and social care) into multidisciplinary neighbourhood groups delivering case manage to high-risk patients	• Co-location of GPs in hospital as part of a complex care hub model • Introduction of new health coach roles in primary care
Information & research	• Data-driven risk stratification approach to initially select high-risk patients, prioritising older people (gradual move away from this approach with experience gained, following general consensus that this was not targeting the ‘right’ patients)	• Data-driven risk stratification approach to initially select high-risk patients, prioritising multimorbid people (gradual move away from this approach with experience gained, following general consensus that this was not targeting the ‘right’ patients)
Technologies & medical products	• Some joining of health and social care records through the Salford Integrated Record	• Plans to join up electronic records, but difficulties in implementing • ‘Patient Knows Best’ online care plan tool to enable self-management • Telehealth management used on a subset of patients by the complex care hub to keep track of vital signs and alert staff to any changes that might require escalation/ follow-up
Financing	• Pump-prime funding by NHS England through Vanguard programme (£5.33 m in 2015/16)^b^• Pooling of health and social care budgets (originally for over 65 s) extending to all adult health and social care services	• Pump-prime funding by NHS England through Vanguard programme (£5.27 m in 2015/16)^c^ • Changed funding for those GP practices incorporated into Symphony Healthcare Services Ltd, with some fund pooling and spending decided on locally • Aim to move towards ‘outcome-based financing’

^a^Intervention focused primarily on those over 65 years old in Salford, and those with multimorbidity in South Somerset – however, this focus is with the aim of having the maximum impact on the measures for the whole population in combination with the other interventions.

^b^Integrated care organisation full business case 2016.^12^

^c^Estimate given by programme director.

## Methods

We sought to evaluate the impact of the entire place-based model of care (the ‘intervention’) on population-level outcomes. The evaluation took place in the context of wider service delivery changes across England, with other sites (our comparator) also commonly targeting high-risk patients using case management approaches (Table 1). This means that any effects measured will mostly be driven by changes other than case management that were introduced, namely the novel, prevention-focused aspects of each intervention site model (plus any scaling up of case management).

### Data

We used two nationally representative sources of data. For measuring patient experience and health status, we used the national General Practitioner Patient Survey (GPPS), a postal survey administered to a sample of registered patients from all GP practices in England annually, which has been conducted twice a year from 2012 until 2016 (annually from 2017).^13^ To assess health care costs, we used the Hospital Episode Statistics (HES) database for England (from April 2009 to March 2018).^14^ This database includes administrative data recording all patient contacts with National Health Service (NHS) hospitals. Using individual-level data from each source, we created a dataset where each observation represents one of eight segments of each GP practice (combinations of presence of multimorbidity, aged over 65 years, and gender) at each time point (by survey wave for GPPS and annually for HES). For example, one row of data represented the outcomes for multimorbid patients, aged 65 years and older, and male (with eight possible unique combinations of these three variables), in GP practice X at time t. We created a dummy for multimorbidity (two or more long-term conditions) for each patient from each data source prior to aggregating the data.

### Outcome measures

For our primary analysis, we measured effectiveness on three outcomes: (1) patient experience, using the question ‘In the last 6 months, have you had enough support from local services or organisations to help you to manage your long-term health condition(s)?’ (GPPS). We analysed the proportion of those with long-term conditions answering, “Yes, definitely”); (2) generic health status (health-related quality of life) as measured by the EQ-5D 5 L index (GPPS),^15^ a continuous measure with 0 equivalent to death and 1 equivalent to perfect health; (3) health care costs, using the total costs of secondary care per registered person per year as calculated by tariff costs (a set of nationally set prices) of each contact,^16^ that is admission, outpatient visit, and emergency department attendance as recorded in the HES database.

We further examined three additional outcomes in secondary analysis to evaluate proxies for the intended ‘prevention’ focus: (4) cost per user of secondary care, that is the total costs of secondary care divided by the count of unique patients contributing to that cost in that year (we replaced the denominator of measure (3) to examine average severity of patients treated in secondary care) (HES); (5) ambulatory care sensitive condition (ACSC)^17^ emergency admissions, that is the number of admissions for conditions considered to be avoidable with appropriate primary care, per list size per year (HES); and (6) primary care utilisation, i.e. the percentage of GPPS respondents reporting that they had seen a GP or nurse in the preceding six months.

### Analysis

We analysed the data using a quasi-experimental difference-in-differences design. We compared GP practices within Salford and South Somerset Vanguard sites (intervention) to control (‘usual care’) practices, using two comparators (excluding practices in other Vanguard sites): (1) all other GP practices in the rest of England; and (2) NHS Rightcare peers. Rightcare peers are the 10 most similar geographical areas, defined by NHS England on the basis of 12 demographic variables.^18^ Identification of the intervention’s causal effect using difference-in-differences analysis assumes that there are parallel trends in outcomes if the intervention was not implemented. This assumption is not testable over the entire analysis period since the intervention has been implemented. However, we assessed the plausibility of this parallel-trends assumption by examining the interaction of intervention status and continuous time in the pre-intervention period.^19^ We employed propensity score weighting proposed by Stuart et al. to ensure comparability of intervention and control units.^20^

We defined the intervention start date as the date when two sites received their first set of Vanguard funding in April 2015, by which date the sites began implementing the respective care model. We used all data available. For GPPS we used three pre-period years (July 2012-March 2015, six survey waves) and two years follow-up (July 2015-April 2017, four survey waves). For HES data we used six pre-period years (April 2009-March 2015) and three years follow-up (April 2015-March 2018). We first report the overall post-period effect before decomposing the effect by year to observe any variation in the effects over time.

It is possible that effects might be diluted at the population level. We therefore also analysed subgroup effects only on those patients with multimorbidity who were likely to be most affected by the intervention. We further tested the robustness of our results to the gradual roll-out or planning effects prior to the intervention to understand the possible effect of the intervention having been implemented before sites received funding. To do so we estimated models where we dropped the year prior to the intervention.

All analyses were conducted in November 2019 using STATA version 15. Further detail on the analysis is presented in the Online Supplement. Propensity scores and inverse probability weighting ensured there were few differences between intervention and control groups based on observable characteristics, and we identified parallel trends in all outcomes in the pre-period (Online supplement).

## Results

Table 2 shows the findings from the regression analysis; estimates are the adjusted intervention effects from the propensity-weighted difference-in-differences models.

**Table 2.** Regression results at the whole population-level.

	(1) Rest of England controls		(2) NHS Rightcare controls	
	n	Adjusted intervention effect (95% CI)^a^	n	Adjusted intervention effect (95% CI)^a^
*Salford*				
Primary outcomes				
Experience (support for long-term conditions)	294 919	0.017 (–0.015 to 0.050)	19,689	0.001 (–0.035 to 0.037)
Health (EQ5D)	297,313	–0.005 (–0.017 to 0.007)	19,822	–0.003 (–0.017 to 0.011)
Cost (total cost of secondary care per registered patient and year)	458,732	73.806*** (37.641 to 109.971)	26,801	136.574*** (62.098 to 211.050)
Secondary outcomes				
Cost per user of secondary care	458,732	138.472*** (76.378 to 200.566)	26,801	156.609** (57.470 to 255.748)
ACSC emergency admissions	458,732	0.001 (–0.002 to 0.004)	26,801	0.001 (–0.003 to 0.005)
Primary care utilisation	297,623	–0.005 (–0.018 to 0.008)	19,846	–0.002 (–0.017 to 0.013)
*South Somerset*				
Primary outcomes				
Experience (support for long-term conditions)	293,919	0.020 (–0.022 to 0.061)	28,635	0.008 (–0.042 to 0.058)
Health (EQ5D)	296,301	–0.007 (–0.022 to 0.008)	28,835	–0.012 (–0.0271 to 0.004)
Cost (total cost of secondary care per registered patient, per year)	457,012	44.545*** (20.351 to 68.739)	40,472	83.773*** (40.966 to 126.579)
Secondary outcomes				
Cost per user of secondary care	457,012	129.717*** (68.256 to 191.178)	40,472	168.385*** (93.876 to 242.893)
ACSC emergency admissions	457,012	0.005*** (0.002 to 0 .007)	40,472	0.004** (0.002 to 0.007)
Primary care utilisation	296,608	0.001 (–0.009 to 0.011)	28,855	0.003 (–0.010 to 0.016)

CI: confidence interval.

***p < 0.001; **p < 0.05; *p < 0.1.

^a^Models adjusted for multimorbidity status, age over 65 years, gender, GP practice size, proportion of GP practice list size that is male, proportion of GP practice list size that is over 75, proportion of male GPs, proportion of non-UK GPs, proportion of GPs aged over 50 years, number of full-time equivalent GPs, index of multiple deprivation, and time and GP practice fixed effects. Standard errors clustered at the GP practice level.

There were no statistically significant differences in intervention sites compared to controls in terms of patient experience or health status as measured by EQ5D. There was however an increase in the costs of secondary care over the post-intervention-period in both intervention sites, at £73.81 per registered patient per year in Salford and £44.55 in South Somerset. There was also an increase in the total cost of secondary care per user by approximately £138 per person per year in Salford and £130 in South Somerset. In South Somerset, there was a statistically significant if small increase in ACSC emergency admissions, at an additional 5 admissions per 1000 registered patients per year. We did not observe any effect on primary care utilisation in either site.

**Figure 1.**
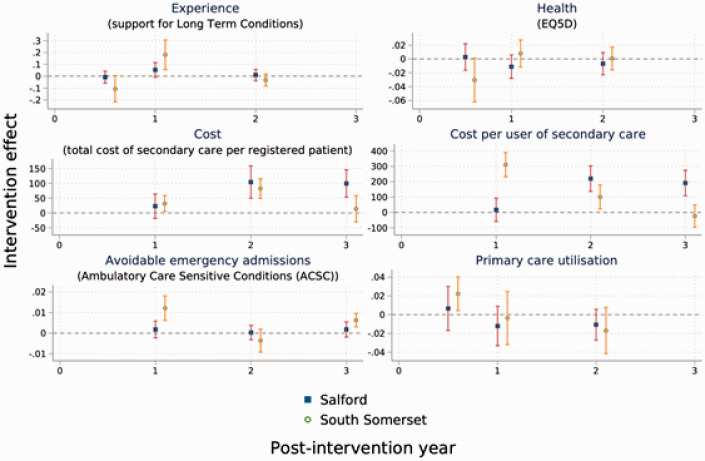
Intervention effect by post-intervention time-point. Note: Measures occurred at both sites at the same time but are displayed at slightly different time points for visual clarity only. ‘Dots’ indicate point estimates, bars indicate 95% confidence intervals. X-Axis values indicate years after implementation (1= one year after implementation, 2 = two years after implementation, 3 = three years after implementation); Year 3 data were available for secondary care outcomes only; GPPS data were not available for year 3.

Figure 1 shows the decomposition of the intervention effect by post-intervention year in each site.

Average intervention effect differed by site and over time. There was a statistically significant increase in patient experience of care in South Somerset at the end of year one and an initial increase in primary care utilisation in the first six months. Similar trends were observed for Salford, but these were not statistically different from control sites. In terms of costs, there were statistically significant increases in Salford in years 2 and 3 for total cost per registered patient and cost per user of secondary care; for South Somerset this effect was only observed for years 1 and 2 post-intervention. As noted above, South Somerset experienced an increase in ACSC emergency admissions, but this effect was limited to year 1 and year 3 post-intervention; there was no discernible trend for Salford.

Repeating the analyses for the subgroup of patients with multimorbidity gave broadly similar results although findings tended to be more attenuated (Online supplement). For example, increases in cost per user were higher compared to the whole population, at £173 in Salford and £268 in South Somerset. We also found that South Somerset had consistently higher total cost of secondary care for multimorbid patients (their primary target group), whereas Salford did not.

Findings also remained broadly similar for models excluding the final year prior to the introduction of Vanguard payment (2014/15) (Online supplement).

## Discussion

This evaluation of two new models of care in England that sought to take a more population health management approach in order to achieve the triple aim found that both sites, on average, experienced an increase in total costs of secondary care and cost per user of secondary care. There were no statistically significant effects on other measured outcomes such as patient experience of care and health status. Cost increases appear to have been driven by increasing average patient severity in hospital. This could be explained by a deliberate aim to keep those with lower need out of hospital, and/or increasingly addressing unmet need. Intervention effects varied by sites, which likely reflects that the two sites implemented a different set of changes at different points in time. There was a more apparent short-term negative effect on costs and ACSC emergency admissions in South Somerset; this might be attributable to more rapid implementation and direct changes to health services.^11^

### Limitations

We used a single intervention starting point to estimate the post-intervention effect, whereas in practice individual service changes were implemented gradually. We chose this time point to reflect Vanguard funding, conducting in effect an ‘intention to treat’ analysis. We allowed intervention effects to vary in the years following the intervention, but it would have been speculative to project these trends further.

A key challenge in any evaluation of complex care models is the identification of an appropriate comparator, in particular where change is being implemented widely in an effort to integrate service delivery as has been the case in England and in many other countries. Our evaluation therefore focused on capturing effects of comparatively ‘novel’ aspects of the care models introduced over the analysis period. We choose three primary outcome measures based on available data and attempting to represent the health system ‘triple aim’.^2^ It is possible that results differ based on a different choice of outcomes. We were unable to capture all system costs for a full cost-effectiveness evaluation.

### Results in context

Our findings are broadly in line with previous evaluations of interventions that targeted populations at high risk of hospitalisation in the UK and internationally. These have similarly identified increased costs with increased access and identification of unmet need.^5^,^21^^–23^ A recent analysis of the South Somerset Vanguard programme was able to study the effects of each individual service delivery intervention using data on directly treated individuals. Similar to our study, that work found an increase in the total costs of primary, community and hospital care while there was no statistically significant effect of (prevention-oriented) enhanced primary care on service utilization.^24^ An analysis of a pooled sample of all 23 population-based Vanguard models also found no significant reduction in emergency admissions over three years in total, although there was some evidence of a relative net reduction compared to controls in the third year.^25^ This points to possible improvements over the longer-term, but the findings are not comparable on the precise measures we used in our analysis.

There is an assumption that wider organisational changes are required to achieve maximum results, in particular cost-savings, of population-based models.^11^ Elsewhere we suggest that national policy and practice barriers might have to be addressed if organisational changes are to be fully implemented in the English system.^26^,^27^ Future research should examine the long-term effects of new care models when affected organisations have had more time to implement change, and examine the optimal combination of interventions to fully ‘unpack the black box’ of what is effective for what outcome.^28^

## Conclusions

Population health management interventions might lead to unintended secondary care cost increases in the short to medium term. Cost increases appear to be driven by average patient severity increases in hospital. Prevention-focused population health management models of integrated care, like previous more targeted models, do not immediately improve the health system’s triple aim.

## Supplementary Material

Supplementary material
