# Supplementary material for: Does prevention-focused integration lead to the triple aim? An evaluation of two new care models in England
Source: J Health Serv Res Policy. 2020 Oct 27;26(2):125–32. doi: 10.1177/1355819620963500 (PMC8013794; doi:10.1177/1355819620963500)
Supplement: Supplementary material [file sj-pdf-1-hsr-10.1177_1355819620963500.pdf]

Online Supplement

Table of Contents

Ambulatory care sensitive conditions ***1***

Further methods details ***2***

Timeline of implementation ***5***

Evaluation of propensity weighting strategy ***6***

Parallel trends tests ***8***

Multimorbidity results ***16***

Robustness results ***19***

Ambulatory care sensitive conditions

Admission for ACSC (from list below) as primary diagnosed condition (Adapted from Harrison Mark J, Dusheiko Mark, Sutton Matt, Gravelle Hugh, Doran Tim, Roland Martin. Effect of a national primary care pay for performance scheme on emergency hospital admissions for ambulatory care sensitive conditions: controlled longitudinal study *BMJ* 2014; 349 :g6423)

**ACSC condition**	**ICD-10**
Asthma	J45, J46
Chronic Ischaemic Heart Disease	I20, I240, I248, I249, I25
Congestive Heart Failure	I110, I130, I50, J81
COPD	J20, J41, J42, J43, J44, J47
Diabetes	E100-108, E110-118, E130-138, E140-148
Epilepsy and convulsions	G40, G41, G568, R568
Hypertensions	I10, I119

**ACSC condition**	**ICD-10**
Diabetes Hypo	E162
Iron-deficiency anaemia	D501, D508, D509
Cellulitis	L03, L04, L080, L088, L089, L88, L980
Dehydration & gastroenteritis	E86, K522, K528, K529
ENT	H66, H67, J02, J03, J04, J06, J312
Gangrene	R02
Nutritional deficiency	E40, E41, E42, E43, E550, E643
Pelvic inflammatory	N70, N73, N74
Perforated/bleeding ulcer	K250-256, K260-262, K264-266, K270-K272, K273-276, K280-282, K284-286
Polynephritis and UTI	N10, N11, N12, N136, N300, N308, N309
Other vaccine preventable diseases	A35, A36, A37, A80, B05, B06, B161, B169, B180, B181, B26, G000, M014

Further methods details

HES data

For HES multimorbidity, we use a count of 30 long-term conditions[17] from ICD-10 codes recorded in inpatient data (multimorbidity status is switched on based on any observed inpatient admission contact and applied to any hospital contact in subsequent years to minimise false zeros).

We take the collapsed sum for each GP practice segment. For comparability, we make the counts per head of registered population by subsequently dividing the size of the registered population in that GP practice segment (age, gender, over 65 status). There are no data available on proportion of multimorbid patients per practice which means that this raw denominator is too large for each segment of our data. We take this into account by further adjusting the denominator using data on

multimorbidity population proportions from the literature,[18] assuming 14% of under 65s have multimorbidity and 67% of over 65s.

To make this adjustment, we use data on proportion of population with multimorbidity from Barnett, K., et al. (2012). "Epidemiology of multimorbidity and implications for health care, research, and medical education: a cross-sectional study." The Lancet 380(9836): 37-43. Within Table 1 of their article they calculate the proportion of each age-range with multimorbidity. We use this proportion plus the absolute number included in each of the age-bands in their data to calculate the overall proportion of multimorbidity expected in those aged over/under 65, i.e. to fit the age cut-offs we use in our data. These proportions are then used to adjust the age, gender, over 65 status population counts for each practice. For example, from Barnett et al. we find that 67% of those over 65 have multimorbidity on average. Therefore, we attribute 67% of the known over 65 population in the given practice at the given time to the multimorbid denominator, and 33% to the non-multimorbid.

We would be concerned if the proportion of multimorbid population was changing differently in our intervention versus control group, as the above adjustment would not be able to account for this. If this were the case, we would observe bias in our estimates caused by changes in composition over time rather than intervention effects. We are able to test whether this compositional change occurs using our GPPS data. We calculate the ratio of survey respondents who are multimorbid:non-multimorbid in each of our other data segments (i.e. age, gender, over 65 status, GP practice, time). We then use this ratio as the outcome in a regression mimicking our main analysis difference-in-difference (adjusting for age, gender, over 65 status, GP practice, and time fixed-effects). We find no significant effect on the interaction of intervention*post for either site suggesting there is a low chance of bias due to multimorbidity compositional changes over time.

GPPS data

For GPPS multimorbidity, we count from self-report data on 15 specified long-term conditions plus we count the option of selecting “another long-term condition” as a single condition.

We use the survey weights to take the weighted mean for each GP practice segment.

Propensity matching

This method proposed by Stuart EA, Huskamp HA, Duckworth K et al. Using propensity scores in difference-in-differences models to estimate the effects of a policy change. Health Serv Outcomes Res Methodol. 2014;14(4):166-82, uses all intervention (post-) and control (pre- and post-) groups and creates analysis weights using observable covariates (multimorbidity status, over 65 status, gender, practice size, proportion of list size that is male, proportion of list size that is over 75, proportion of male GPs, proportion of non-UK GPs, proportion of GPs over 50, number of full-time equivalent GPs, index of multiple deprivation) and pre-intervention outcomes to reflect comparability to the intervention group in the pre-intervention period.

Regression models

All regression models are estimated using adjusted ordinary least squares, including time and GP practice fixed effects, whether the practice segment represents multimorbid/non-multimorbid, gender and over/under 65 age groups, and additional time-varying GP practice co-variates. We cluster standard errors at the GP practice level, the level on which we assign intervention participation.

We use a single post-intervention period for each site, effectively employing an ‘intention to treat’ policy analysis at the population-level to evaluate the effectiveness of national policymakers incentivising the roll-out of this type of model.

Timeline of implementation

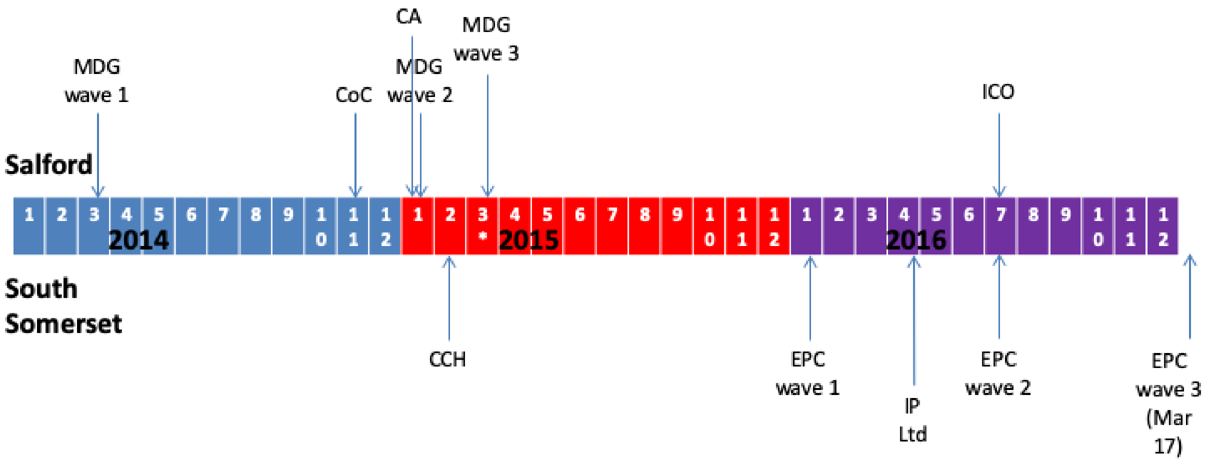

*Note: MDG = Multi-disciplinary group; CoC = Centre of Contact; CA = Community Assets; ICO = Integrated Care Organisation; CCH = Complex Care Hub; EPC = Enhanced Primary Care; IP Ltd = formation of a Ltd company of Integrated GP Practices. * = Vanguard status awarded to both sites*

Evaluation of propensity weighting strategy

HES data

**Salford**	Treated		Control		Treated - weighted		Control - weighted		St diff	St diff weighted
	Mean	SD	Mean	SD	Mean	SD	Mean	SD		
GP list size	5086.73	3323.86	6795.92	4162.52	5122.77	3312.35	5101.29	3052.61	-0.41	0.01
Proportion of list size male	0.51	0.03	0.50	0.02	0.51	0.03	0.51	0.03	0.17	0.01
Proportion of GPs non-uk	0.43	0.44	0.35	0.41	0.42	0.44	0.43	0.42	0.19	-0.01
Proportion of GPs over 50	0.56	0.41	0.57	0.37	0.57	0.40	0.56	0.39	-0.03	0.02
Proportion of male GPs	0.62	0.30	0.57	0.27	0.62	0.30	0.61	0.31	0.18	0.03
Number of full-time equivalent GPs	2.90	2.13	4.38	3.12	2.94	2.14	2.94	2.13	-0.47	0.00
Proportion of list size over 75	0.06	0.05	0.07	0.03	0.06	0.02	0.06	0.03	-0.33	-0.01
Index of multiple deprivation (IMD)	42.24	19.55	26.13	17.09	42.55	19.59	42.37	19.96	0.94	0.01

**South Somerset**	Treated		Control		Treated - weighted		Control - weighted		St diff	St diff weighted
	Mean	SD	Mean	SD	Mean	SD	Mean	SD		
GP list size	6992.04	3571.00	6795.92	4162.52	7096.86	3561.04	7067.77	3880.18	0.05	0.01
Proportion of list size male	0.49	0.01	0.50	0.02	0.49	0.01	0.49	0.02	-0.65	0.04
Proportion of GPs non-uk	0.12	0.24	0.35	0.41	0.10	0.16	0.10	0.18	-0.57	0.01
Proportion of GPs over 50	0.55	0.38	0.57	0.37	0.54	0.36	0.53	0.37	-0.05	0.03
Proportion of male GPs	0.55	0.18	0.57	0.27	0.56	0.17	0.56	0.23	-0.10	0.01
Number of full-time equivalent GPs	4.93	2.28	4.38	3.12	4.95	2.29	4.90	3.17	0.18	0.02
Proportion of list size over 75	0.10	0.03	0.07	0.03	0.10	0.03	0.10	0.04	0.79	-0.06
Index of multiple deprivation (IMD)	17.66	9.93	26.13	17.09	17.07	9.40	17.03	11.89	-0.50	0.00

GPPS data

**Salford**	Treated		Control		Treated - weighted		Control - weighted		St. diff	St. diff weighted
	Mean	SD	Mean	SD	Mean	SD	Mean	SD		
GP list size	5334.30	3522.82	7076.83	4289.28	5516.26	3509.62	5542.10	3494.93	-0.41	-0.01
Proportion of list size male	0.51	0.03	0.50	0.02	0.51	0.02	0.51	0.03	0.27	0.00
Proportion of GPs non-uk	0.45	0.45	0.37	0.44	0.44	0.45	0.44	0.43	0.16	0.00
Proportion of GPs over 50	0.47	0.40	0.49	0.36	0.44	0.38	0.43	0.36	-0.05	0.02
Proportion of male GPs	0.60	0.31	0.55	0.27	0.60	0.30	0.60	0.30	0.20	0.02
Number of full-time equivalent GPs	3.05	2.37	4.42	3.19	3.26	2.41	3.26	2.34	-0.43	0.00
Proportion of list size over 75	0.06	0.06	0.08	0.03	0.06	0.04	0.07	0.03	-0.40	-0.01
Index of multiple deprivation (IMD)	41.76	19.52	26.11	17.14	42.21	19.24	41.99	20.26	0.91	0.01

**South Somerset**	Treated		Control		Treated - weighted		Control - weighted		St. diff	St. diff weighted
	Mean	SD	Mean	SD	Mean	SD	Mean	SD		
GP list size	6912.96	3474.93	7076.83	4289.28	7125.02	3551.08	7133.26	3950.31	-0.04	0.00
Proportion of list size male	0.49	0.01	0.50	0.02	0.49	0.01	0.49	0.02	-0.65	0.02
Proportion of GPs non-uk	0.14	0.31	0.37	0.44	0.13	0.27	0.12	0.22	-0.53	0.01
Proportion of GPs over 50	0.49	0.40	0.49	0.36	0.49	0.37	0.48	0.37	0.02	0.01
Proportion of male GPs	0.51	0.21	0.55	0.27	0.52	0.20	0.51	0.23	-0.15	0.01
Number of full-time equivalent GPs	4.63	2.13	4.42	3.19	4.71	2.17	4.70	2.87	0.07	0.01
Proportion of list size over 75	0.10	0.03	0.08	0.03	0.10	0.03	0.10	0.04	0.74	-0.01
Index of multiple deprivation (IMD)	18.53	10.69	26.11	17.14	17.94	10.16	17.95	12.76	-0.44	0.00

Parallel trends tests

**Salford**<br><br>(p-score reported for interaction of intervention status and continuous time in the pre-intervention period)					
Outcome	Without weight	Weighted	Rightcare weighted	Multimorbidity weighted	Multimorbidity Rightcare weighted
Cost (total cost of secondary care per registered patient, per year)	0.1412	0.5338	0.0700	0.1214	0.7792
ACSC emergency admissions	0.0157	0.4927	0.1367	0.9416	0.1342
Cost per user (cost/by count of unique persons using secondary care in that year)	0.1686	0.7877	0.2727	0.8852	0.3251
Health (EQ5D)	0.0531	0.977	0.6954	0.5634	0.3403
Experience (support for LTCs)	0.9472	0.8101	0.9182	0.782	0.6665
Primary care (% reporting seeing a GP/nurse in previous 6 months)	0.4875	0.6044	0.6385	0.0532	0.1695

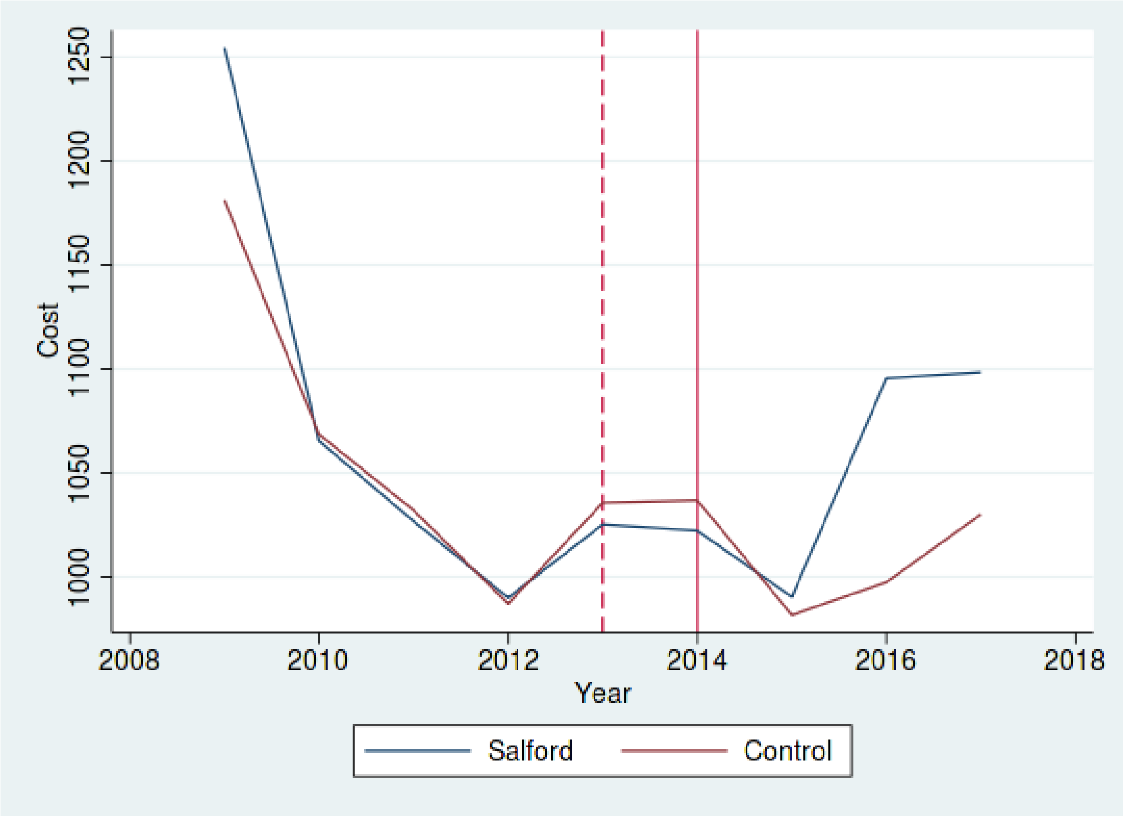

Cost
1250
1200
1150
1100
1050
1000
2008
2010
2012
2014
2016
2018
Year
Salford
Control

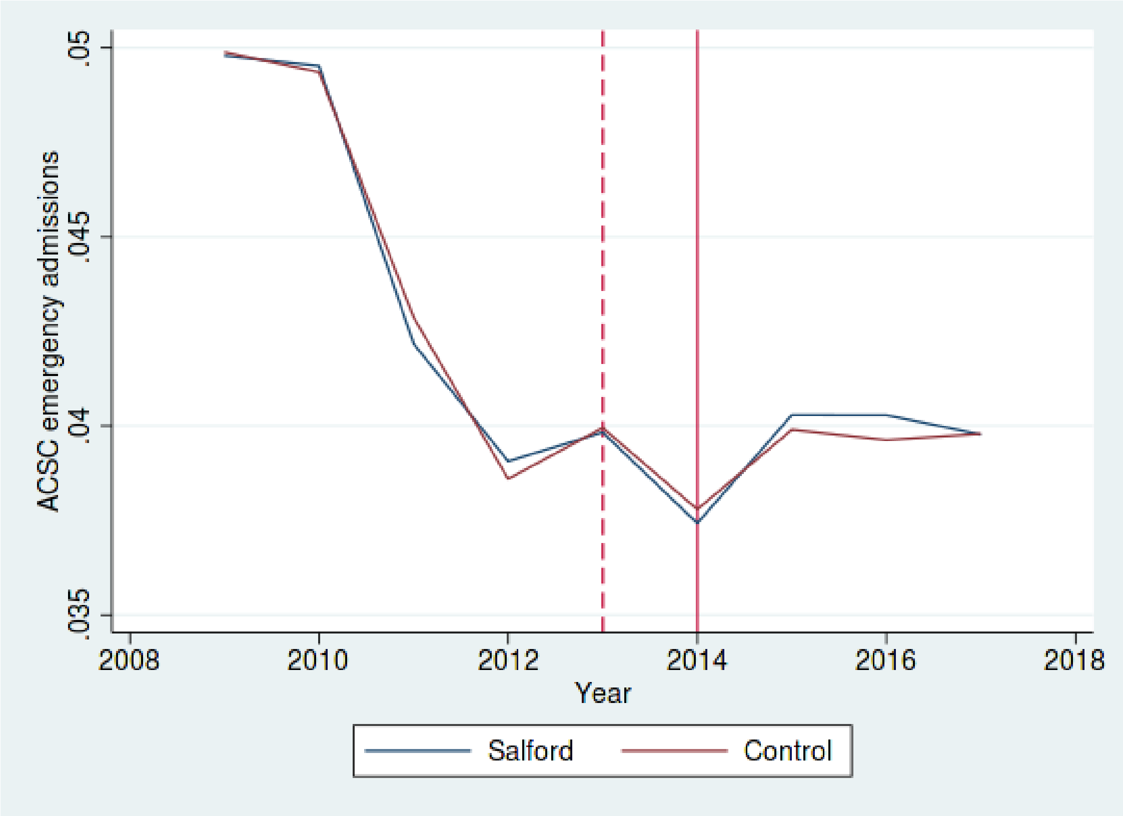

ACSC emergency admissions
.05
.045
.04
.035
2008
2010
2012
2014
2016
2018
Year
Salford
Control

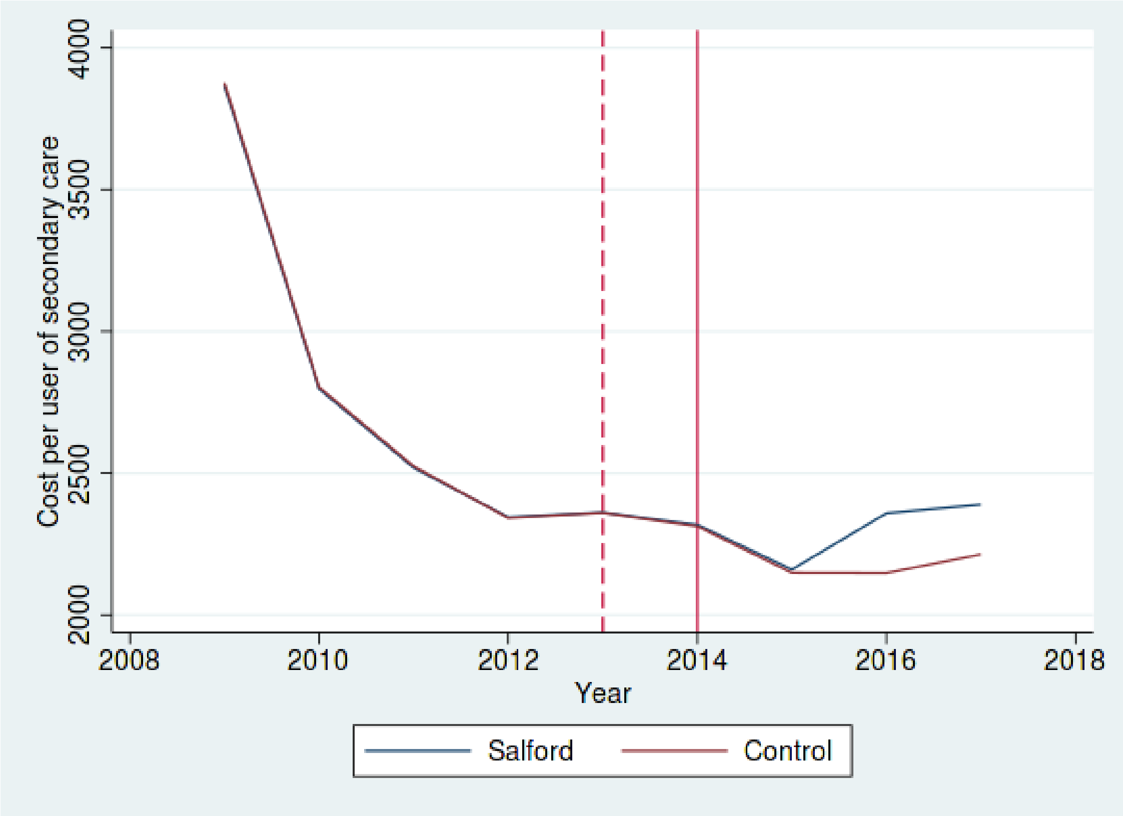

Cost per user of secondary care
2000
2500
3000
3500
4000
2008
2010
2012
2014
2016
2018
Year
Salford
Control

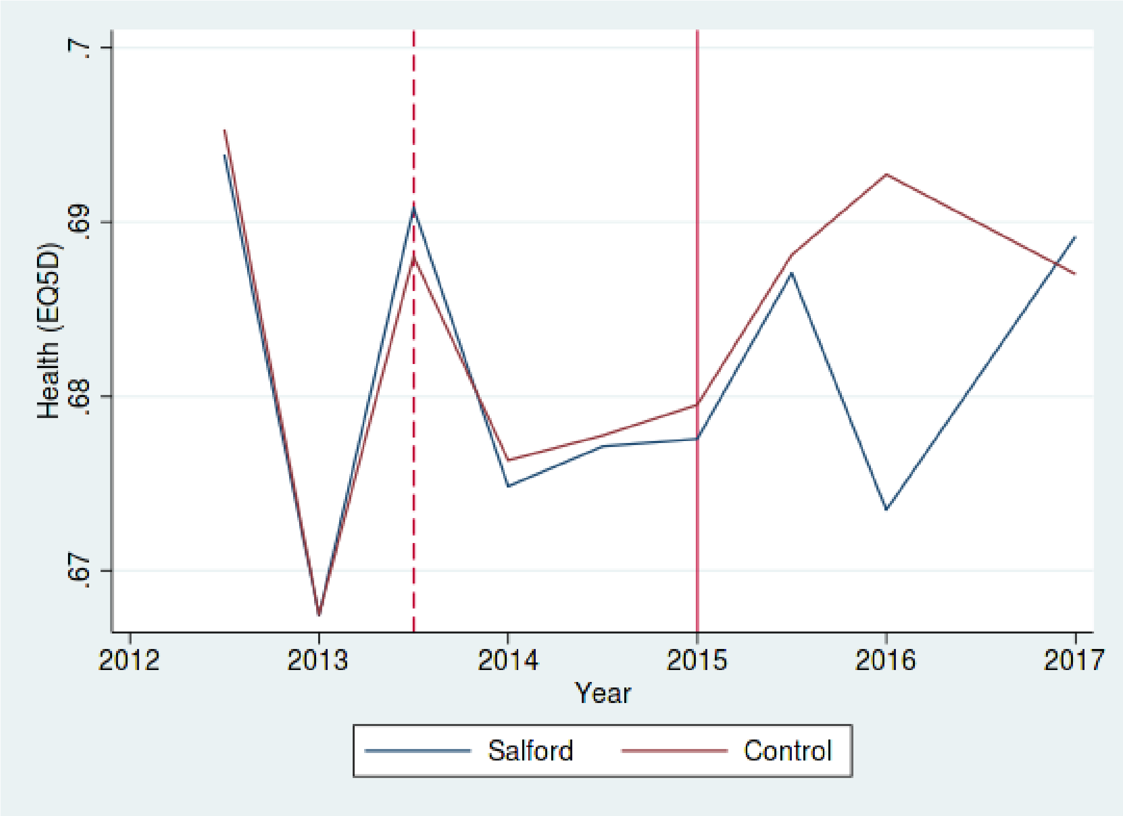

Health (EQ5D)
.67
.68
.69
.7
2012
2013
2014
2015
2016
2017
Year
Salford
Control

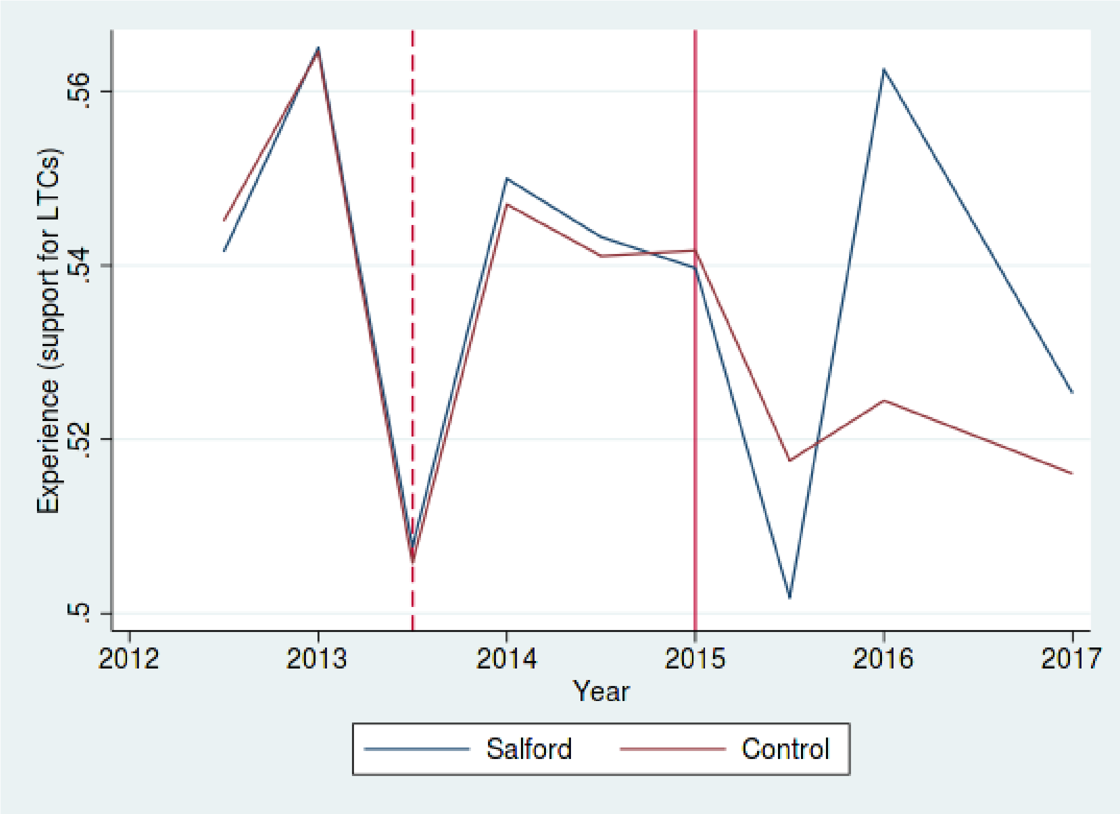
Experience (support for LTCs)
.5
.52
.54
.56
2012
2013
2014
2015
2016
2017
Year
Salford
Control

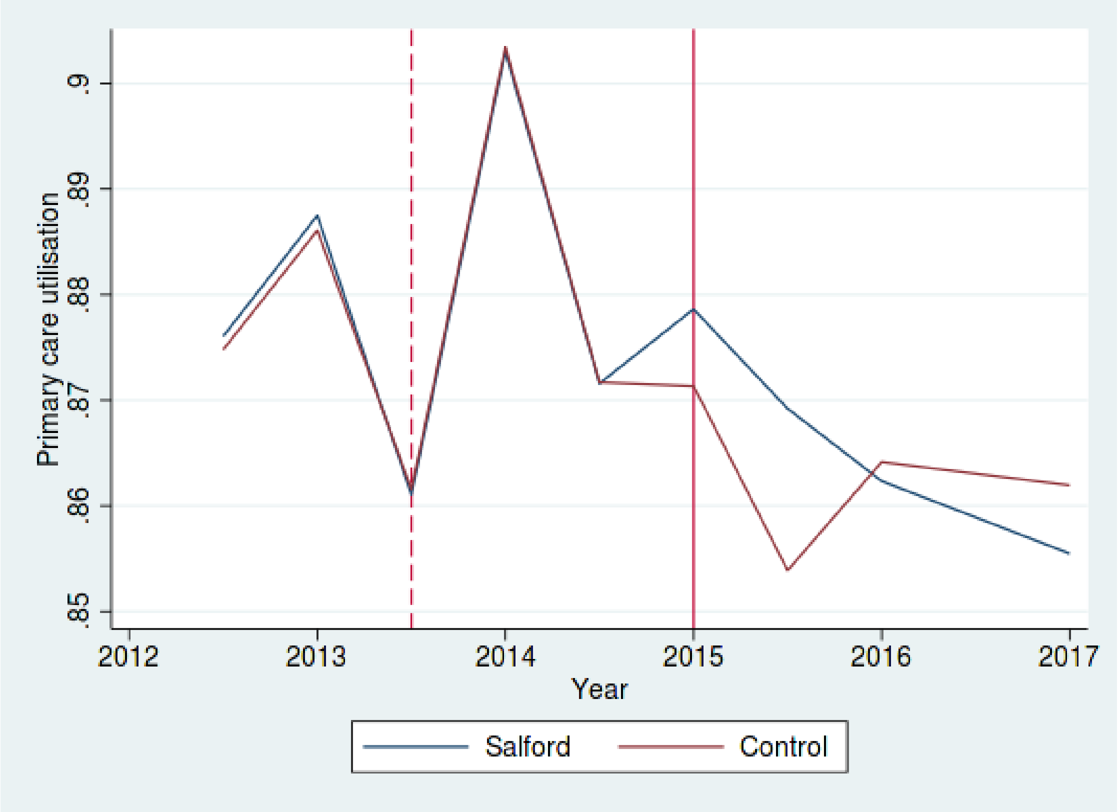
Primary care utilisation
.85
.86
.87
.88
.89
.9
2012
2013
2014
2015
2016
2017
Year
Salford
Control

**South Somerset**<br><br>(p-score reported for interaction of intervention status and continuous time in the pre-intervention period)					
Outcome	Without weight	Weighted	Rightcare weighted	Multimorbidity weighted	Multimorbidity Rightcare weighted
Cost (total cost of secondary care per registered patient, per year)	0.6256	0.8452	0.9471	0.0988	0.1570
ACSC emergency admissions	0.2568	0.5736	0.8801	0.3255	0.7537
Cost per user (cost/by count of unique persons using secondary care in that year)	0.3444	0.9516	0.9084	0.9341	0.9243
Health (EQ5D)	0.8216	0.8608	0.7208	0.9173	0.8735
Experience (support for LTCs)	0.3395	0.7278	0.6655	0.6734	0.752
Primary care (% reporting seeing a GP/nurse in previous 6 months)	0.9208	0.7177	0.7045	0.1677	0.2978

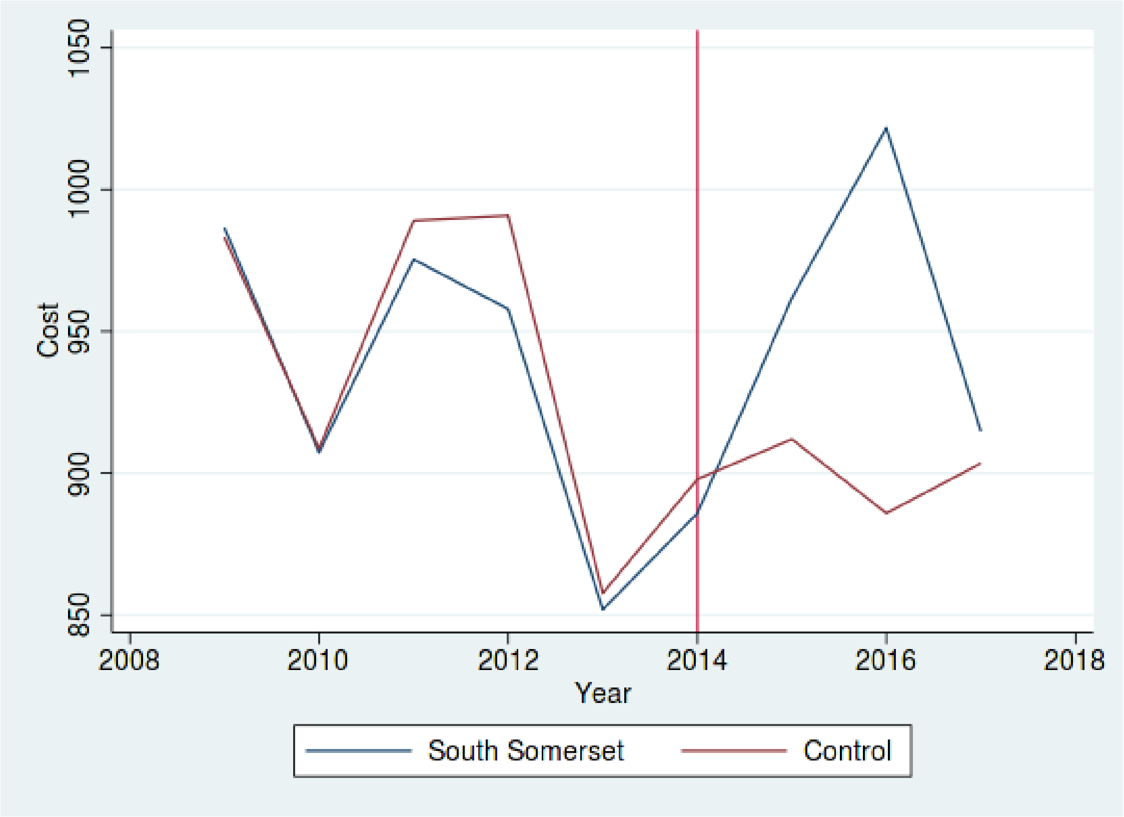
Cost
850
900
950
1000
1050
2008
2010
2012
2014
2016
2018
Year
South Somerset
Control

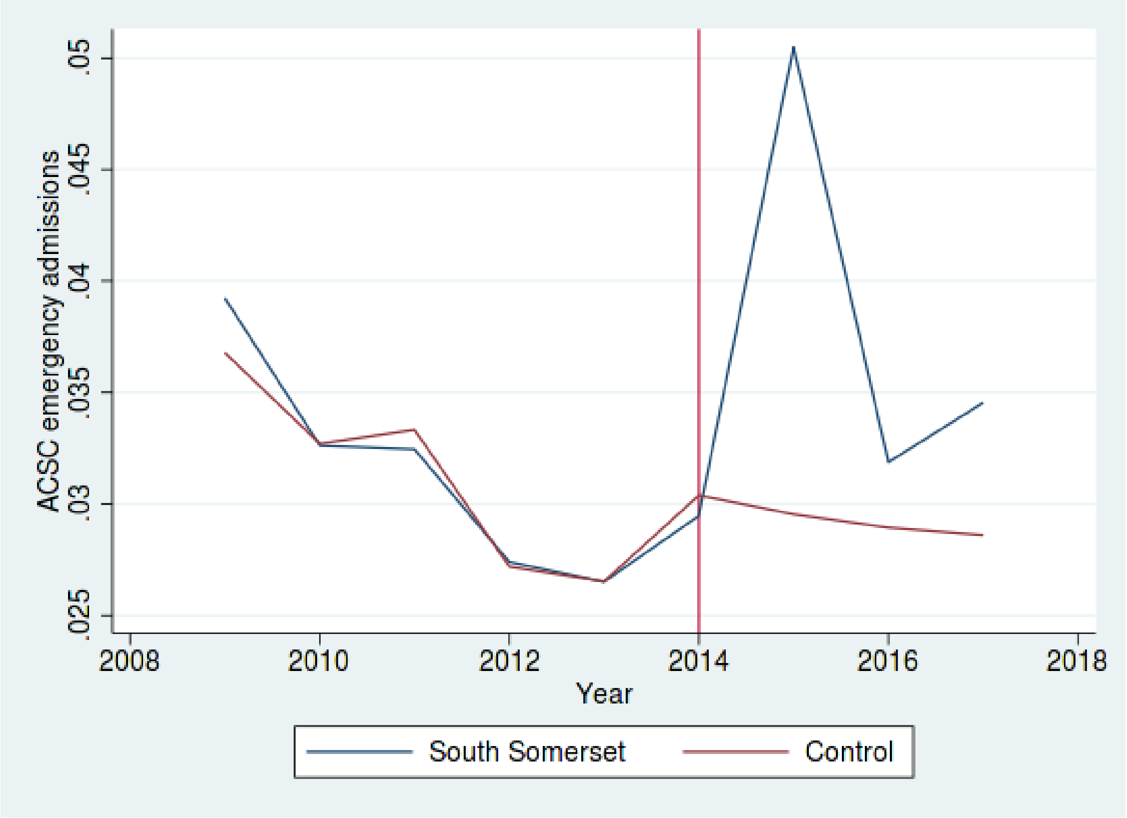
ACSC emergency admissions
.025
.03
.035
.04
.045
.05
2008
2010
2012
2014
2016
2018
Year
South Somerset
Control

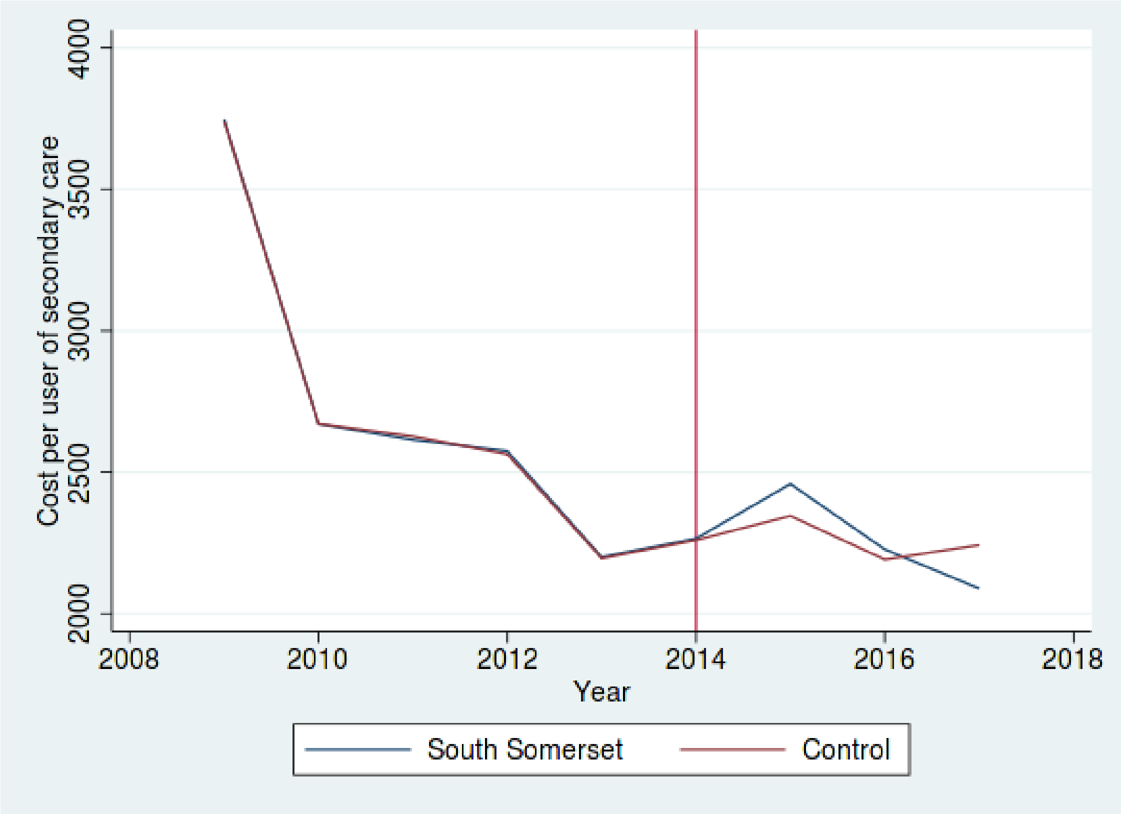
Cost per user of secondary care
2000
2500
3000
3500
4000
2008
2010
2012
2014
2016
2018
Year
South Somerset
Control

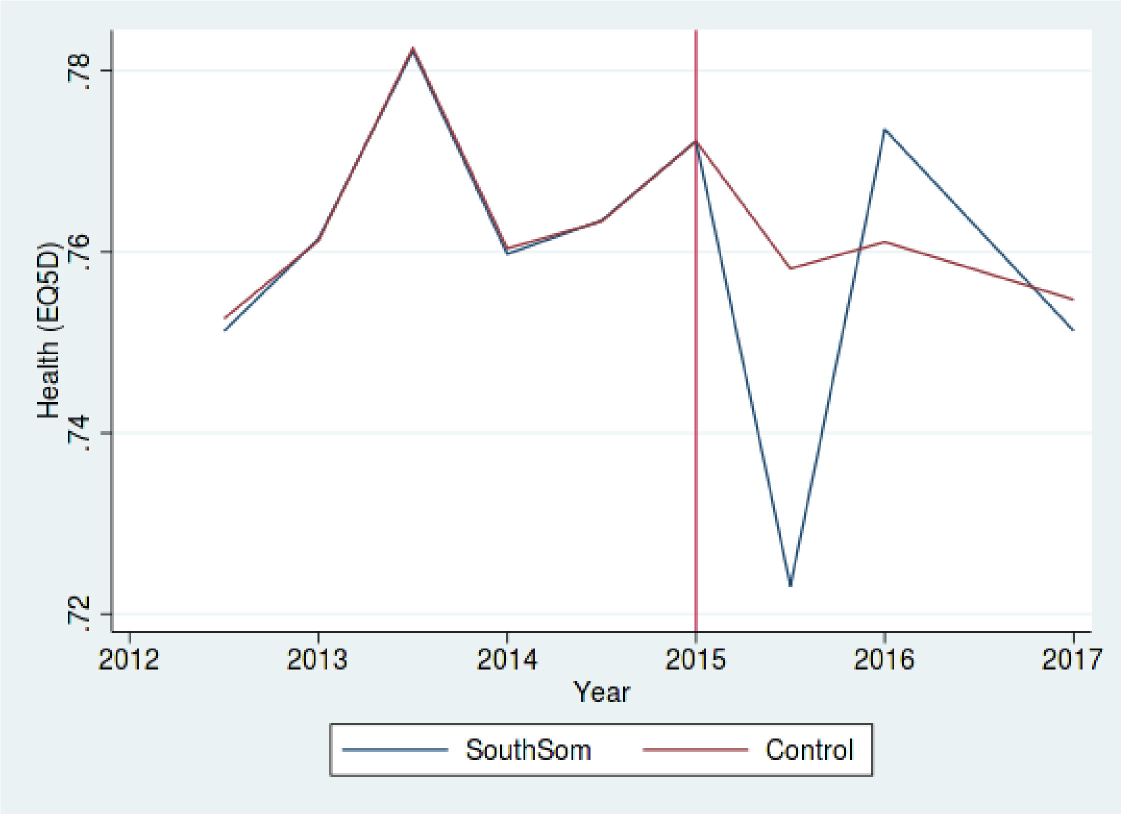
Health (EQ5D)
.72
.74
.76
.78
2012
2013
2014
2015
2016
2017
Year
SouthSom
Control

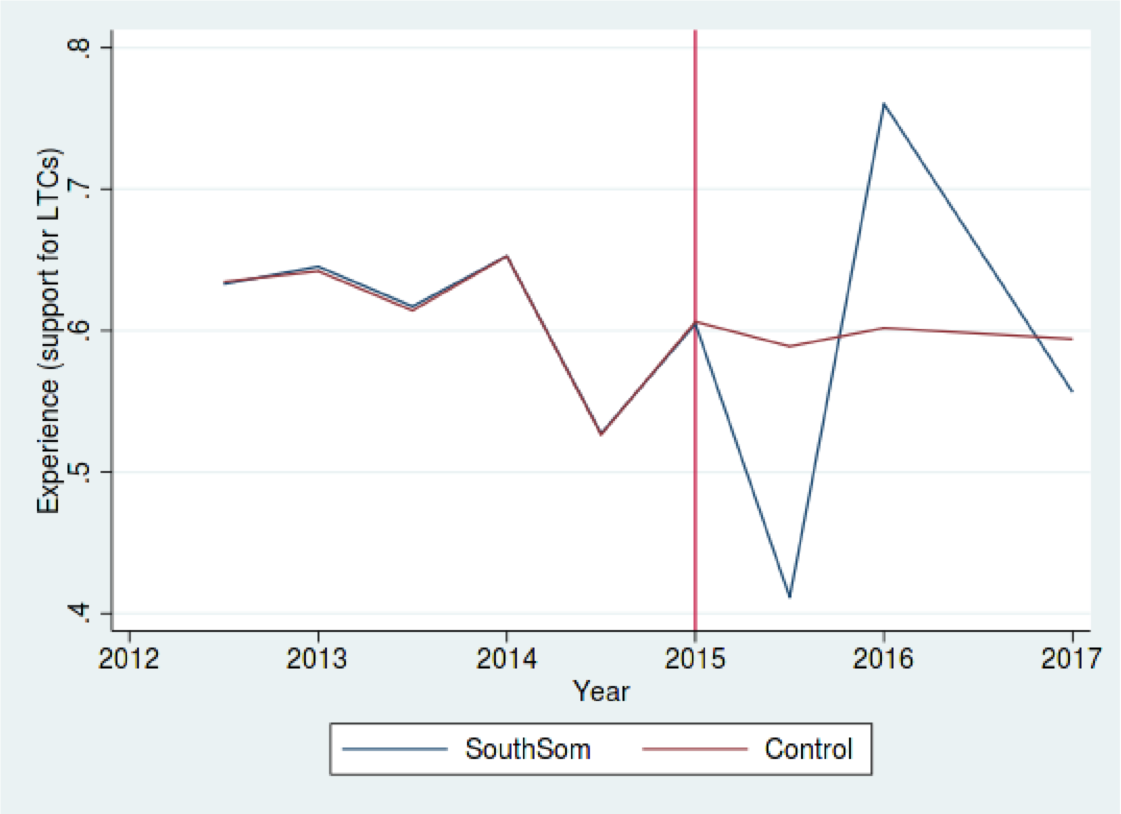
Experience (support for LTCs)
.4
.5
.6
.7
.8
2012
2013
2014
2015
2016
2017
Year
SouthSom
Control

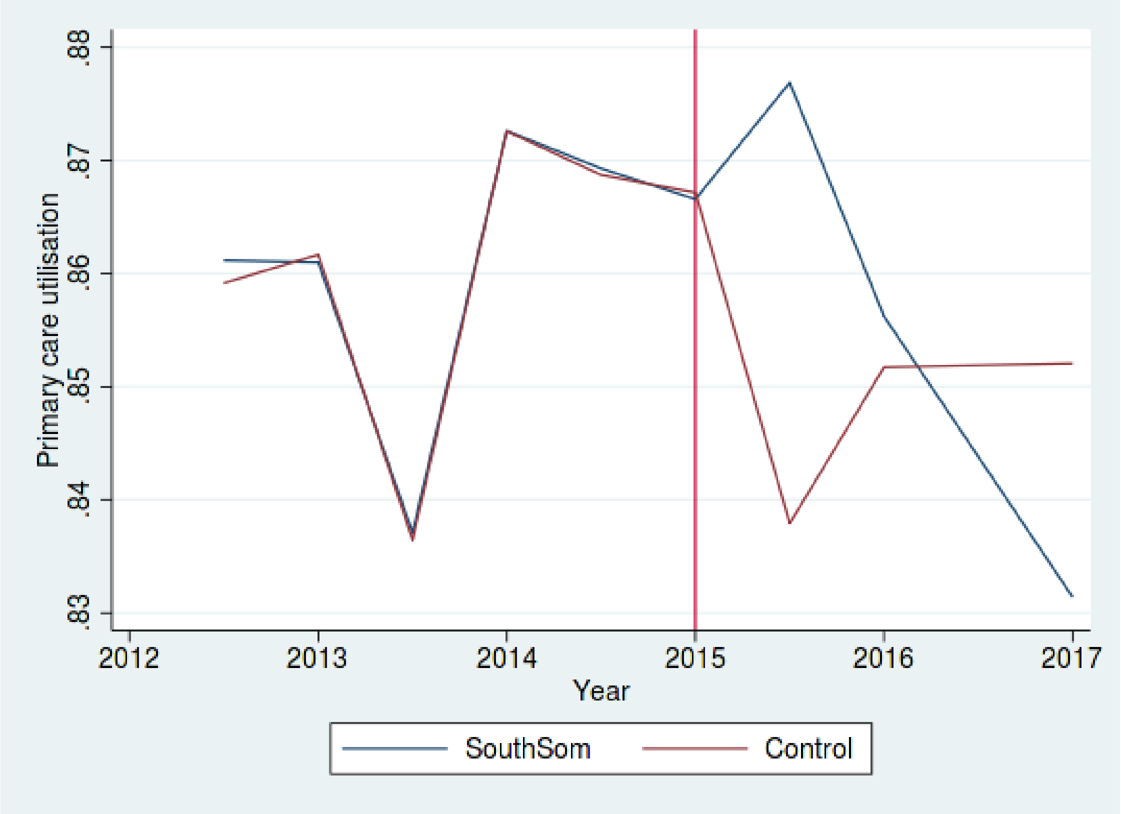
Primary care utilisation
.83
.84
.85
.86
.87
.88
2012
2013
2014
2015
2016
2017
Year
SouthSom
Control

Multimorbidity results

	(1) Rest of England controls		(2) NHS Rightcare controls	
**Salford**	n	Adjusted# intervention effect (95% CI)	n	Adjusted# intervention effect (95% CI)
Primary outcomes				
Experience (support for LTCs)	152,299	0.037*	11,060	0.018
		(-0.003 to 0.076)		(-0.027 to 0.063)
Health (EQ5D)	152,906	-0.013	11,084	-0.01
		(-0.035 to 0.009)		(-0.035 to 0.015)
Cost (total cost of secondary care per registered patient, per year)	229,226	26.674	13,389	101.026*
		(-31.596 to 84.943)		(-4.099 to 206.151)
Secondary outcomes				
Cost per user (cost/by count of unique persons using secondary care in that year)	229,226	173.356**	13,389	209.216**
		(53.651 to 293.062)		(22.293 to 396.138)
ACSC emergency admissions	229,226	0.0001	13,389	0.003
		(-0.004 to 0.005)		(-0.005 to 0.011)
Primary care (% reporting seeing a GP/nurse in previous 6 months)	153,182	0.003	11,107	0.005
		(-0.013 to .019)		(-0.013 to 0.024)
**South Somerset**				
Primary outcomes				
Experience (support for LTCs)	151,695	-0.003	13,749	-0.014
		(-0.074 to 0.067)		(-0.090 to 0.062)
Health (EQ5D)	152,299	-0.015	13,799	-0.026
		(-0.046 to 0.015)		(-0.059 to 0.006)
Cost (total cost of secondary care per registered patient, per year)	228,366	80.791***	20,236	103.619***
		(44.842 to 116.740)		(57.543 to 149.696)
Secondary outcomes				
Cost per user (cost/by count of unique persons using secondary care in that year)	228,366	267.689***	20,236	328.628***
		(150.843 to 384.535)		(184.760to 472.495)

ACSC emergency admissions	228,366	0.008***	20,236	0.006**
		(0.032 to 0.012)		(0.0004 to 0.012)
Primary care (% reporting seeing a GP/nurse in previous 6 months)	152,573	-0.001	13,819	0.002
		(-0.012 to 0.010)		(-0.012 to 0.016)

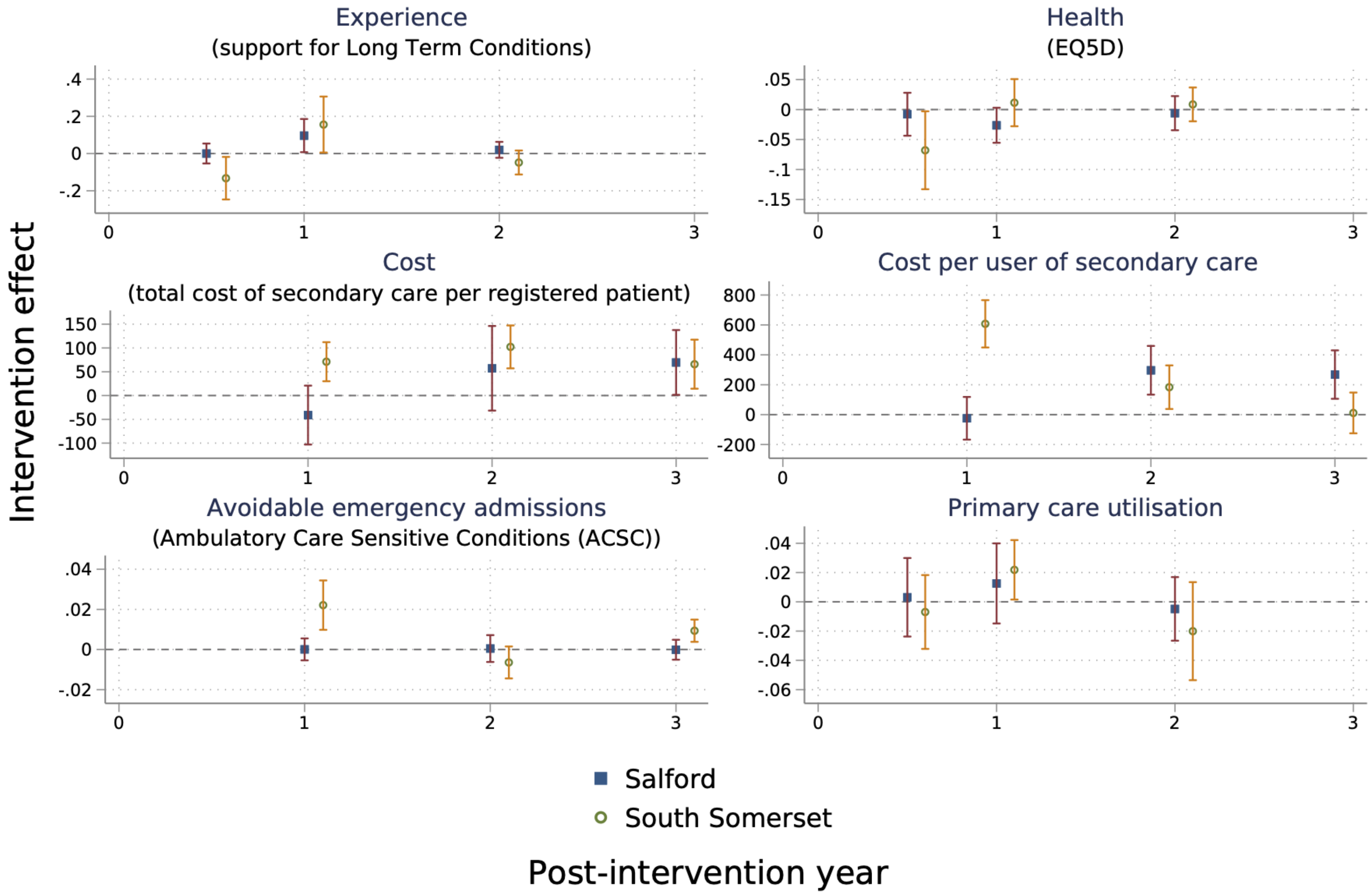
Experience
(support for Long Term Conditions)
Health
(EQ5D)
Cost
(total cost of secondary care per registered patient)
Cost per user of secondary care
Avoidable emergency admissions
(Ambulatory Care Sensitive Conditions (ACSC))
Primary care utilisation
Intervention effect
Salford
South Somerset
Post-intervention year

Robustness results

Population models

	Main models reported in main paper - Weighted				Robustness check	
**Salford**	(1) Rest of England controls		(2) NHS Rightcare controls		(3) Prior year (2014/15) dropped	
Primary outcomes	n	Adjusted# intervention effect (95% CI)	n	Adjusted# intervention effect (95% CI)	n	Adjusted# intervention effect (95% CI)
Experience (support for LTCs)	294,919	0.017	19,689	0.001	261,369	0.027*
		(-0.015 to 0.050)		(-0.035 to 0.037)		(-0.005 to 0.060)
Health (EQ5D)	297,313	-0.005	19,822	-0.003	264,436	-0.015**
		(-0.017 to 0.007)		(-0.017 to 0.011)		(-0.027 to -0.003)
Cost (total cost of secondary care per registered patient, per year)	458,732	73.806***	26,801	136.574***	407,317	82.181***
		(37.641 to 109.971)		(62.098 to 211.050)		(44.043 to 120.320)
Secondary outcomes						
Cost per user (cost/by count of unique persons using secondary care in that year)	458,732	138.472***	26,801	156.609**	407,317	171.239***
		(76.378 to 200.566)		(57.470 to 255.748)		(101.014 to 241.464)

ACSC emergency admissions	458,<br>732	0.001	26,8<br>01	0.001	407,<br>317	0.002
		(-0.002 to 0.004)		(-0.003 to 0.005)		(-0.001 to 0.005)
Primary care (% reporting seeing a GP/nurse in previous 6 months)	297,<br>623	-0.005	19,8<br>46	-0.002	264,<br>837	-0.002
		(-0.018 to 0.008)		(-0.017 to 0.013)		(-0.015 to 0.011)
**South Somerset**						
Primary outcomes						
Experience (support for LTCs)	293,<br>919	0.020	28,6<br>35	0.008	260,<br>468	0.012
		(-0.022 to 0.061)		(-0.042 to 0.058)		(-0.029 to 0.053)
Health (EQ5D)	296,<br>301	-0.007	28,8<br>35	-0.012	263,<br>519	-0.003
		(-0.022 to 0.008)		(-0.0271 to 0.004)		(-0.019 to 0.013)
Cost (total cost of secondary care per registered patient, per year)	457,<br>012	44.545***	40,4<br>72	83.773***	405,<br>805	46.491***
		(20.351 to 68.739)		(40.966 to 126.579)		(18.025 to 74.958)
Secondary outcomes						
Cost per user (cost/by count of unique persons using secondary care in that year)	457,<br>012	129.717***	40,4<br>72	168.385***	405,<br>805	160.067***

		(68.256 to 191.178)		(93.876 to 242.893)		(88.483to 237.650)
ACSC emergency admissions	457,012	0.005***	40,472	0.004**	405,805	0.005***
		(0.002 to 0 .007)		(0.002 to 0.007)		(0.003 to 0.008)
Primary care (% reporting seeing a GP/nurse in previous 6 months)	296,608	0.001	28,855	0.003	263,917	-0.002
		(-0.009 to 0.011)		(-0.010 to 0.016)		(-0.012 to .009)

Multimorbid patient models

Main model - Weighted	Main models reported in main paper				Robustness check	
	(1) Rest of England controls		(2) NHS Rightcare controls		(3) Prior year (2014/15) dropped	
**Salford**	n	Adjusted# intervention effect (95% CI)	n	Adjusted# intervention effect (95% CI)	n	Adjusted# intervention effect (95% CI)
Primary outcomes						
Experience (support for LTCs)	152,299	0.037*	11,060	0.018	134,644	0.046**
		(-0.003 to 0.076)		(-0.027 to 0.063)		(0.001 to 0.091)

Health (EQ5D)	152,906	-0.013	11,084	-0.01	135,400	-0.031**
		(-0.035 to 0.009)		(-0.035 to 0.015)		(-0.051 to -0.010)
Cost (total cost of secondary care per registered patient, per year)	229,226	26.674	13,389	101.026*	203,520	35.657
		(-31.596 to 84.943)		(-4.099 to 206.151)		(-26.317 to 97.631)
Secondary outcomes						
Cost per user (cost/by count of unique persons using secondary care in that year)	229,226	173.356**	13,389	209.216**	203,520	234.069**
		(53.651 to 293.062)		(22.293 to 396.138)		(97.015 to 371.122)
ACSC emergency admissions	229,226	0.0001	13,389	0.003	203,520	0.0004
		(-0.004 to 0.005)		(-0.005 to 0.011)		(-0.005 to 0.005)
Primary care (% reporting seeing a GP/nurse in previous 6 months)	153,182	0.003	11,107	0.005	135,753	0.008
		(-0.013 to .019)		(-0.013 to 0.024)		(-0.010 to 0.026)
**South Somerset**						
Primary outcomes						
Experience (support for LTCs)	151,695	-0.003	13,749	-0.014	134,105	-0.03
		(-0.074 to 0.067)		(-0.090 to 0.062)		(-0.111 to 0.050)
Health (EQ5D)	152,299	-0.015	13,799	-0.026	134,857	0.001

		(-0.046 to 0.015)		(-0.059 to 0.006)		(-0.031 to 0.032)
Cost (total cost of secondary care per registered patient, per year)	228,366	80.791***	20,236	103.619***	202,764	82.199***
		(44.842 to 116.740)		(57.543 to 149.696)		(41.184 to 123.214)
Secondary outcomes						
Cost per user (cost/by count of unique persons using secondary care in that year)	228,366	267.689***	20,236	328.628***	202,764	320.711***
		(150.843 to 384.535)		(184.760 to 472.495)		(170.428 to 470.995)
ACSC emergency admissions	228,366	0.008***	20,236	0.006**	202,764	0.009***
		(0.032 to 0.012)		(0.0004 to 0.012)		(0.004 to 0.014)
Primary care (% reporting seeing a GP/nurse in previous 6 months)	152,573	-0.001	13,819	0.002	135,208	-0.005
		(-0.012 to 0.010)		(-0.012 to 0.016)		(-0.021 to 0.010)
